# The Association Between Head Trauma and BPPV: A Nested Case-Control Study Using a National Health Screening Cohort

**DOI:** 10.3390/diagnostics15172171

**Published:** 2025-08-27

**Authors:** Dae Myoung Yoo, Bo-Ram Yang, Kyeongmin Han, Hyo Geun Choi, Goun Choe, Jin Woong Choi, Bong Jik Kim

**Affiliations:** 1 Hallym Data Science Laboratory, College of Medicine, Hallym University, Anyang 14068, Republic of Korea; ydm1285@naver.com (D.M.Y.); hankm1130@naver.com (K.H.); 2 College of Pharmacy, Chungnam National University, Daejeon 34134, Republic of Korea; br.yang@g.cnu.ac.kr; 3 Department of Bio-AI Convergence, Chungnam National University, Daejeon 34134, Republic of Korea; 4 Suseoseoulent Clinic, Seoul 06349, Republic of Korea; pupen@naver.com; 5 Department of Otolaryngology-Head and Neck Surgery, College of Medicine, Chungnam National University, Chungnam National University Sejong Hospital, Daejeon 35015, Republic of Korea; gounchoemd@gmail.com; 6 Department of Otorhinolaryngology-Head and Neck Surgery, College of Medicine, Chungnam National University, Chungnam National University Hospital, Daejeon 35015, Republic of Korea; choijw@cnu.ac.kr; 7 Brain Research Institute, College of Medicine, Chungnam National University, Daejeon 35015, Republic of Korea

**Keywords:** benign paroxysmal positional vertigo, risk factor, head trauma, nested case–control study

## Abstract

**Background/Objectives**: Benign paroxysmal positional vertigo (BPPV) is one of the most common vestibular disorders and is characterized by transient but very severe vertigo, increasing fall risk, especially in older people. While many risk factors have been reported, there are still contradicting papers and evidence from large-scale studies remains limited. **Methods**: This nationwide, nested case–control study utilized Korean National Health Insurance Service-Health Screening Cohort data to investigate possible risk factors for BPPV. In particular, it examined the association between prior head trauma and BPPV, proposing prior head trauma as a plausible and clinically relevant risk factor. From an initial cohort of 514,866 participants, 29,467 BPPV cases were matched 1:4 with 117,868 controls based on age, sex, income, region, and index date. Conditional logistic regression, with overlap weighting, assessed the risk of BPPV associated with head trauma and other potential factors. **Results**: Head trauma was modestly more prevalent in the BPPV group (2.29% vs. 1.83%) and was significantly associated with BPPV (adjusted OR 1.28, 95% CI 1.17–1.40, *p* < 0.001). The corresponding Absolute Risk Increase (ARI) was 0.82 percentage points over the entire follow-up and 0.66 percentage points within 1 year. The association persisted across most subgroups including both demographic and clinical factors except underweight individuals and those with high comorbidity scores. **Conclusions**: This large-scale analysis reinforces head trauma as a significant risk factor for BPPV, providing population-level evidence that may guide clinical assessment and prevention strategies.

## 1. Introduction

Benign paroxysmal positional vertigo (BPPV) is a common vestibular disorder characterized by sudden, brief spells of vertigo occurring with head movements [1,2,3,4,5]. These vertigo spells can significantly impair daily functioning, causing imbalance, unsteadiness, and an increased risk of falls, which is particularly concerning among older adults [6,7]. BPPV is reported to account for up to 20–30% of cases seen in dizziness specialty clinics and contributes to nearly 9% of all patients presenting with vertigo symptoms in primary care settings [8]. The estimated lifetime prevalence of BPPV ranges from 2.4% to 3.2%, making it one of the most frequent causes of peripheral dizziness in clinical practice [9]. Given its high prevalence and potential for causing falls and injury, BPPV imposes a considerable burden both on affected individuals and on healthcare systems.

Understanding the risk factors for disease is crucial in the diagnosis, management and prevention of the disease. Several risk factors for BPPV have been identified in the literature [4,10,11,12]. Increasing age has been consistently identified as a risk factor, and BPPV has been reported to be approximately twice as common in women as in men [13]. Other conditions associated with BPPV include head trauma, osteoporosis or low bone mineral density, vestibular migraine, prolonged bed rest, and certain metabolic disorders such as diabetes mellitus and hyperlipidemia. A two- to- three-fold increased risk of BPPV was reported in patients with osteopenia or osteoporosis, compared with individuals with normal bone mineral density [14]. These associations suggest that both mechanical and metabolic factors may underlie the pathogenesis of BPPV.

Nevertheless, the literature presents conflicting results regarding several risk factors for BPPV, and controversies persist about their relative significance. Considering both the prevalence and potential serious consequences of BPPV, including the risk of falls and reduced quality of life, revisiting and clarifying the risk factors of BPPV using large-scale data could provide critical clinical insights.

Given this, our nationwide, large-scale study aims to investigate the associations between various potential risk factors—including head trauma, smoking, BMI, and alcohol consumption—and the occurrence of BPPV. By leveraging comprehensive national health insurance data and employing robust statistical methodologies, especially including exact matching and overlap weighting, we aim to generate more precise, evidence-based perspectives to support clinical decision-making and direct future research.

## 2. Materials and Methods

### 2.1. Ethics

The study was approved by the ethics committee of Hallym University (2022-12-005). The requirement for written informed consent was waived by the Institutional Review Board. All procedures were conducted in accordance with the guidelines and regulations of the Hallym University Ethics Committee.

### 2.2. Exposure (Head Trauma)

Head trauma was defined as a diagnosis of ICD-10 codes S00 to S09 by neurologists, neurosurgeons, or emergency medicine doctors at least twice, confirmed by CT assessments of the head and neck (Claim codes: HA401-HA416, HA441-HA443, HA451-HA453, HA461-HA463, or HA471-HA473). Only head trauma events occurring before the index date were included.

### 2.3. Outcome (Benign Paroxysmal Vertigo)

BPPV was selected based on ICD-10 codes (BPPV: H811) by neurologist or otolaryngologist. To enhance diagnostic accuracy, we included only participants who had visited the clinics at least twice with a diagnosis of BPPV.

### 2.4. Study Population and Participant Selection

Comprehensive information on the Korean National Health Insurance Service-Health Screening Cohort (KNHIS-HEALS) data is available in a previous publication [15]. In brief, data were obtained from the KNHIS-HEALS, a nationwide cohort of Korean adults aged 40–69 years who participated in health screening between 2002 and 2003 and were followed through 2019 [16,17].

BPPV participants were selected from 514,866 participants with 895,300,177 medical claim codes from 2002 through 2019 (*n* = 30,219). The control group was included if participants were not defined as BPPV from 2002 through 2019 (*n* = 484,647). BPPV participants were excluded for 1 year of washout period (*n* = 750). Control participants were excluded if the participants were diagnosed with BPPV (ICD-10 codes: H811) once (*n* = 22,125). Participants with BPPV were matched to controls at a 1:4 ratio according to age, sex, income, and region of residence. To reduce selection bias, the control participants were sorted using a random number order and were selected from top to bottom. For analysis, the index date of each control was aligned with that of the corresponding BPPV case.

**Figure 1:**
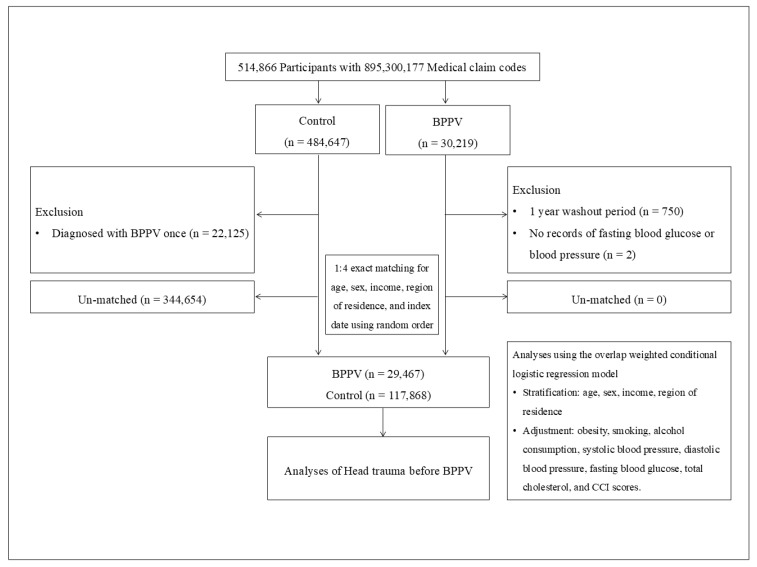
A schematic illustration of the participant selection process that was used in the present study. Of a total of 514,866 participants, 29,467 of BPPV participants were matched with 117,868 of control participants for age, sex, income, and region of residence.

During the matching procedure, a total of 344,654 potential controls were excluded. Ultimately, 29,467 participants with BPPV were matched at a 1:4 ratio with 117,868 controls (Figure 1).

### 2.5. Covariates

Age groups were divided into 5-year intervals: 40–44…, and 85+ years old. A total of 10 age groups were specified. Income groups were classified as 5 classes (class 1 [lowest income]-5 [highest income]). The region of residence was grouped into urban (Seoul, Busan, Daegu, Incheon, Gwangju, Daejeon, and Ulsan) and rural (Gyeonggi-do, Gangwon-do, Chungcheongbuk-do, Chungcheongnam-do, Jeollabuk-do, Jeollanam-do, Gyeongsangbuk-do, Gyeongsangnam-do, and Jeju-do) areas.

Tobacco smoking was classified according to smoking status as nonsmoker, past smoker, or current smoker. Alcohol consumption was categorized by frequency into <1 time per week and ≥1 time per week. Obesity was assessed using body mass index (BMI, kg/m^2^), and BMI was stratified as <18.5 (underweight), ≥18.5 to <23 (normal), ≥23 to <25 (overweight), ≥25 to <30 (obese I), and ≥30 (obese II), based on the Asia–Pacific criteria established by the Western Pacific Regional Office (WPRO) in 2000 [18]. Measurements included systolic and diastolic blood pressure (mmHg), fasting blood glucose (mg/dL), and total cholesterol (mg/dL).

The Charlson Comorbidity Index (CCI) was employed to quantify disease burden based on 17 comorbid conditions [19]. Each participant received a weighted score reflecting both the number and severity of comorbidities. The CCI was treated as a continuous variable ranging from 0 (no comorbidity) to 29 (multiple comorbidities).

### 2.6. Statistical Analyses

To compare general characteristics between the BPPV and control groups, standardized differences were calculated (Table 1).

To estimate the odds ratios (ORs) with 95% confidence intervals (CIs) for the association between head trauma and BPPV, conditional logistic regression was performed in groups matched for age, sex, income, and region of residence. Both the Crude model and Overlap weighted model were applied (Table 2). The 95% confidence interval (CI) was calculated. Additionally, subgroup analyses according to all covariate variable were performed.

We calculated the absolute risk increase (ARI) for BPPV incidence to provide an absolute measure of the excess risk associated with head trauma.

All statistical analyses were conducted using SAS version 9.4 (SAS Institute Inc., Cary, NC, USA). Two-tailed tests were applied, and statistical significance was set at *p* < 0.05. The were used for statistical analyses.

## 3. Results

A total of 514,866 participants were initially included in the study. After applying exclusion criteria, such as single BPPV diagnosis for control group, insufficient washout period, and missing data for fasting blood glucose or blood pressure, the cohort was narrowed down. Subsequently, 1:4 exact matching was performed based on age, sex, income, region of residence, and index date, resulting in a final analysis population of 29,467 participants in the BPPV group and 117,868 in the control group (Figure 1).

Table 1 summarizes the baseline characteristics of the study participants. Slight differences were observed between the BPPV and control groups regarding obesity, smoking use, and alcohol consumption. Among clinical variables, head trauma occurs slightly more often in the BPPV group (2.29% vs. 1.83%), suggesting a potential clinical link. Aside from these variables, the two groups were otherwise very well matched across demographic and clinical factors.

Although the absolute difference in prevalence of head trauma between two groups is small (0.46%), the difference in rates (2.29% vs. 1.83%) is real and biologically and clinically relevant in that head trauma can dislodge otoconia in the inner ear, potentially causing BPPV [20]. Thus, we moved to further investigate whether prior head trauma increases the odds of developing BPPV or not. Logistic regression analysis showed that prior head trauma was significantly associated with increased odds of BPPV by 28% (adjusted odds ratios = 1.28, 95% CI 1.17–1.40, *p* < 0.001) (Table 2).

As an additional measure to complement the odds ratio, we calculated the Absolute Risk Increase (ARI) using the original cohort dataset. Over the entire follow-up period (mean 85.6 ± 55.2 months), the ARI for BPPV associated with head trauma was 0.82 percentage points (4.48% vs. 3.66%), and within 1 year after the index date, the ARI was 0.66 percentage points (1.33% vs. 0.67%) (Supplementary Table S1).

Nearly all subgroups in Table 2 show significantly increased odds of BPPV in patients with history of head trauma except underweight and CCI score = 1 subgroups. Specifically, the association is consistent across age, sex, income, residence, and clinical subgroup factors including smoking, alcohol consumption, blood pressure, glucose, and cholesterol, etc. Especially strong association with prior head trauma was observed in younger patients (Age < 65 years old), people without comorbidities and people with normal/ overweight groups (Table 2).

## 4. Discussion

Prior research has demonstrated a link between head trauma and BPPV, indicating that mechanical displacement of otoconia due to trauma can trigger vertigo episodes [9,21,22,23,24]. Meanwhile, animal studies have demonstrated that acute disruption of the inner ear ultrastructure may underlie the pathogenesis of traumatic BPPV [25]. Interestingly, BPPV following head trauma has also been reported to most commonly involve the posterior canal, as in idiopathic cases [22,26]. Our study provides compelling evidence that prior head trauma is an independent risk factor for BPPV, reinforcing its mechanistic role in the pathophysiology of the condition. Although the prevalence of head trauma was only modestly higher in the BPPV group compared to controls (2.29% vs. 1.83%) as shown in Table 1, this seemingly small difference proved clinically significant. As demonstrated in Table 2, individuals with a history of head trauma before the index date had a significantly increased likelihood of developing BPPV, with an adjusted odds ratio of approximately 1.28. This association remained consistent across various subgroups defined by age, sex, income, and clinical characteristics, although it appeared somewhat weaker in underweight individuals and those with a higher burden of comorbidities. Taken together, these findings confirm that even a modest increase in head trauma prevalence can translate into a meaningful risk for BPPV, underscoring the importance of considering a history of head trauma during the clinical assessment of patients presenting with vertigo.

Beyond head trauma, we also observed differences between the BPPV and control groups in factors such as smoking status, underweight status, and alcohol consumption. Regarding smoking, the evidence linking it to BPPV remains inconclusive. Some earlier studies have examined smoking as a potential risk factor for vestibular disorders in general, but results have been inconsistent. For instance, Sunami et al. (2006) reported no significant association between smoking and BPPV occurrence or recurrence [27]. Similarly, a meta-analysis did not identify smoking as a significant independent risk factor for BPPV [28], highlighting the need for further large-scale research to clarify any potential relationship. Underweight status has not been directly identified as a risk factor for BPPV in the existing literature. However, it may be indirectly relevant due to its potential association with low bone mineral density, which has been linked to BPPV in previous studies [14,29]. Similarly, alcohol consumption has not been specifically established as a direct risk factor for BPPV, although it is known to affect balance and vestibular function. Our large-scale, nationwide study provides a valuable opportunity to investigate these potential associations with greater statistical power. Although our data suggest that these lifestyle factors may not be independent risk factors for BPPV, but modest or indirect effects cannot be entirely excluded. These analyses could contribute to filling knowledge gaps in the literature.

Our study is distinguished by several strengths. First, we utilized a large, nationwide cohort, which enhances the statistical power to detect meaningful associations and reduces selection bias inherent in smaller clinical series. Second, our study employed a rigorous 1:4 exact matching approach based on age, sex, income, region of residence, and index date, as well as overlap weighting, a method designed to optimize covariate balance between groups and minimize residual confounding [30]. This methodological strength is evident in the well-matched baseline characteristics shown in Table 1. Third, unlike many previous studies focusing only on small clinical samples, we were able to evaluate head trauma as a potential independent risk factor for BPPV in a general population setting, with comprehensive subgroup analyses to explore effect modification. Collectively, these features provide robust, population-level evidence to clarify the epidemiological relationship between head trauma and BPPV. This comprehensive approach helps clarify the epidemiological association and provides a stronger evidence base for clinicians and researchers

Despite the strengths of our large, nationwide cohort and rigorous matching methods, our study has several limitations. Although head trauma was significantly associated with BPPV, the overall prevalence was low, raising the possibility of rare-event bias, especially in subgroup analyses. The use of claims data introduces potential misclassification of both BPPV and head trauma diagnoses, and residual confounding cannot be entirely excluded [31], whereas our case definition requiring both a relevant ICD-10 diagnosis and CT confirmation likely improved diagnostic accuracy but may have excluded milder trauma cases, potentially underestimating the incidence. Another limitation is that in this study, the time interval between head trauma and BPPV onset was not considered; only the temporal order of the two conditions was ensured. Therefore, further studies incorporating interval length are warranted. Lastly, the extrapolation of our results to non-Korean populations may be restricted by potential ethnic and healthcare system differences. Furthermore, when interpreting findings from large-scale health insurance claims data or other big data sources, odds ratios may be prone to being overstated due to the large sample size and inherent limitations of such datasets. Thus, our results should be interpreted with caution, and future research is needed to confirm whether these statistical associations translate into meaningful clinical or public health risks.

In summary, our study adds valuable population-based evidence to the understanding of BPPV, highlighting the role of head trauma as a significant factor associated with its development. By leveraging a large national cohort and robust matching methods, we provide precise risk estimates that may inform clinical assessment and patient counseling. Although future research will likely utilize even larger and more precise datasets, along with more sophisticated statistical techniques, to further clarify these associations, our study provides a meaningful contribution to the current understanding of BPPV, while emphasizing the importance of considering prior head trauma in patients presenting with vertigo symptoms and these insights may ultimately contribute to improved prevention strategies and earlier diagnosis of BPPV.

## Figures and Tables

**Table 1:** General Characteristics of Participants.

Characteristics		Total Participants		
		BPPV	Control	Standardized Difference
Age (years old) (*n*, %)				0.00
	40–44	170 (0.60)	680 (0.60)	
	45–49	1398 (4.90)	5592 (4.90)	
	50–54	3374 (11.83)	13,496 (11.83)	
	55–59	5070 (17.78)	20,280 (17.78)	
	60–64	5283 (18.53)	21,132 (18.53)	
	65–69	4913 (17.23)	19,652 (17.23)	
	70–74	4000 (14.03)	16,000 (14.03)	
	75–79	2743 (9.62)	10,972 (9.62)	
	80–84	1192 (4.18)	4768 (4.18)	
	85+	368 (1.29)	1472 (1.29)	
Sex (*n*, %)				0.00
	Male	10,502 (36.83)	42,008 (36.83)	
	Female	18,009 (63.17)	72,036 (63.17)	
Income (*n*, %)				0.00
	1 (lowest)	4621 (16.21)	18,484 (16.21)	
	2	3422 (12.00)	13,688 (12.00)	
	3	4225 (14.82)	16,900 (14.82)	
	4	6091 (21.36)	24,364 (21.36)	
	5 (highest)	10,152 (35.61)	40,608 (35.61)	
Region of residence (*n*, %)				0.00
	Urban	12,937 (45.38)	51,748 (45.38)	
	Rural	15,574 (54.62)	62,296 (54.62)	
Obesity † (*n*, %)				0.09
	Underweight	568 (1.99)	2951 (2.59)	
	Normal	9458 (33.17)	40,057 (35.12)	
	Overweight	8035 (28.18)	30,634 (26.86)	
	Obese I	9509 (33.35)	36,386 (31.91)	
	Obese II	941 (3.30)	4016 (3.52)	
Smoking status (*n*, %)				0.14
	Nonsmoker	22,509 (78.95)	87,659 (76.86)	
	Past smoker	3612 (12.67)	13,077 (11.47)	
	Current smoker	2390 (8.38)	13,308 (11.67)	
Alcohol consumption (*n*, %)				0.07
	<1 time a week	22,433 (78.68)	86,328 (75.70)	
	≥1 time a week	6078 (21.32)	27,716 (24.30)	
Systolic blood pressure (Mean, SD)		126.26 (15.88)	126.82 (16.53)	0.04
Diastolic blood pressure (Mean, SD)		77.38 (10.10)	77.68 (10.45)	0.03
Fasting blood glucose (Mean, SD)		100.55 (25.63)	101.65 (28.34)	0.04
Total cholesterol (Mean, SD)		198.69 (38.57)	199.07 (38.89)	0.01
CCI score (Mean, SD)		0.98 (1.58)	0.95 (1.65)	0.02
Head trauma (*n*, %)		653 (2.29)	2082 (1.83)	0.04

Abbreviations: CCI, Charlson comorbidity index;. † Obesity (BMI, body mass index, kg/m^2^) was categorized as <18.5 (underweight), ≥18.5 to <23 (normal), ≥23 to <25 (overweight), ≥25 to <30 (obese I), and ≥30 (obese II).

**Table 2:** Crude and adjusted odd ratios of Head trauma for BPPV when participants are diagnosed with Head trauma before index date.

Characteristics		N of BPPV	N of Control	Odd Ratios for BPPV (95% Confidence Interval)			
		(Exposure/Total, %)	(Exposure/Total, %)	Crude	*p*-Value	Overlap Weighted Model †	*p*-Value
Total (*n* = 142,555)							
	No Head trauma	27,858/28,511 (97.7%)	111,962/114,044 (98.2%)	1 1		1	
	Head trauma	653/28,511 (2.3%)	2082/114,044 (1.8%)	1.26 (1.15–1.38)	<0.001 *	1.28 (1.17–1.40)	<0.001 *
Age < 65 years old (*n* = 76,475)							
	No Head trauma	15,033/15,295 (98.3%)	60,431/61,180 (98.8%)	1 1		1	
	Head trauma	262/15,295 (1.7%)	749/61,180 (1.2%)	1.41 (1.22–1.62)	<0.001 *	1.43 (1.24–1.65)	<0.001 *
Age ≥ 65 years old (*n* = 66,080)							
	No Head trauma	12,825/13,216 (97.0%)	51,531/52,864 (97.5%)	1		1	
	Head trauma	391/13,216 (3.0%)	1333/52,864 (2.5%)	1.18 (1.05–1.32)	0.005 *	1.20 (1.07–1.34)	0.002 *
Men (*n* = 52,510)							
	No Head trauma	10,212/10,502 (97.2%)	41,039/42,008 (97.7%)	1		1	
	Head trauma	290/10,502 (2.8%)	969/42,008 (2.3%)	1.20 (1.05–1.37)	0.007 *	1.23 (1.08–1.41)	0.002 *
Women (*n* = 90,045)							
	No Head trauma	17,646/18,009 (98.0%)	70,923/72,036 (98.5%)			1	
	Head trauma	363/18,009 (2.0%)	1113/72,036 (1.6%)	1.31 (1.16–1.48)	<0.001 *	1.32 (1.17–1.49)	<0.001 *
Low income (*n* = 61,340)							
	No Head trauma	11,977/12,268 (97.6%)	48,124/49,072 (98.1%)	1		1	
	Head trauma	291/12,268 (2.4%)	948/49,072 (1.9%)	1.23 (1.08–1.41)	0.002 *	1.26 (1.10–1.44)	0.001 *
High income (*n* = 81,215)							
	No Head trauma	15,881/16,243 (97.8%)	63,838/64,972 (98.3%)	1		1	
	Head trauma	362/16,243 (2.2%)	1134/64,972 (1.8%)	1.28 (1.14–1.45)	<0.001 *	1.30 (1.15–1.46)	<0.001 *
Urban residents (*n* = 64,685)							
	No Head trauma	12,667/12,937 (97.9%)	50,878/51,748 (98.3%)	1		1	
	Head trauma	270/12,937 (2.1%)	870/51,748 (1.7%)	1.25 (1.09–1.43)	0.002 *	1.27 (1.11–1.46)	0.001 *
Rural residents (*n* = 77,870)							
	No Head trauma	15,191/15,574 (97.5%)	61,084/62,296 (98.1%)	1		1	
	Head trauma	383/15,574 (2.5%)	1212/62,296 (2.0%)	1.27 (1.13–1.43)	<0.001 *	1.28 (1.14–1.44)	<0.001 *
Underweight (*n* = 3519)							
	No Head trauma	558/568 (98.2%)	2874/2951 (97.4%)	1		1	
	Head trauma	10/568 (1.8%)	77/2951 (2.6%)	0.67 (0.34–1.30)	0.237	0.69 (0.35–1.35)	0.274
Normal weight (*n* = 49,515)							
	No Head trauma	9226/9458 (97.6%)	39,275/40,057 (98.1%)	1		1	
	Head trauma	232/9458 (2.5%)	782/40,057 (2.0%)	1.26 (1.09–1.46)	0.002 *	1.29 (1.11–1.50)	0.001 *
Overweight (*n* = 38,669)							
	No Head trauma	7847/8035 (97.7%)	30,104/30,634 (98.3%)	1		1	
	Head trauma	188/8035 (2.3%)	530/30,634 (1.7%)	1.36 (1.15–1.61)	<0.001 *	1.38 (1.16–1.63)	<0.001 *
Obese (*n* = 50,852)							
	No Head trauma	10,227/10,450 (97.9%)	39,709/40,402 (98.3%)	1		1	
	Head trauma	223/10,450 (2.1%)	693/40,402 (1.7%)	1.25 (1.07–1.46)	0.004 *	1.25 (1.07–1.45)	0.001 *
Non-smoker (*n* = 110,168)							
	No Head trauma	22,026/22,509 (97.9%)	86,165/87,659 (98.3%)	1		1	
	Head trauma	483/22,509 (2.2%)	1494/87,659 (1.7%)	1.26 (1.14–1.40)	<0.001 *	1.26 (1.14–1.40)	<0.001 *
Past smoker and current smoker (*n* = 32,387)							
	No Head trauma	5832/6002 (97.2%)	25,797/26,385 (97.8%)	1		1	
	Head trauma	170/6002 (2.8%)	588/26,385 (2.2%)	1.28 (1.08–1.52)	0.005 *	1.32 (1.11–1.57)	0.002 *
Alcohol consumption < 1 time a week (*n* = 108,761)							
	No Head trauma	21,964/22,433 (97.9%)	84,908/86,328 (98.4%)	1		1	
	Head trauma	469/22,433 (2.1%)	1420/86,328 (1.6%)	1.28 (1.15–1.42)	<0.001 *	1.27 (1.14–1.41)	<0.001 *
Alcohol consumption ≥ 1 time a week (*n* = 33,794)							
	No Head trauma	5894/6078 (97.0%)	27,054/27,716 (97.6%)	1		1	
	Head trauma	184/6078 (3.0%)	662/27,716 (2.4%)	1.28 (1.08–1.51)	0.004 *	1.30 (1.10–1.53)	0.002 *
SBP < 140 mmHg and DBP < 90 mmHg (*n* = 39,816)							
	No Head trauma	7845/8020 (97.8%)	31,236/31,796 (98.2%)	1		1	
	Head trauma	175/8020 (2.2%)	560/31,796 (1.8%)	1.24 (1.05–1.48)	0.013 *	1.26 (1.06–1.50)	0.009 *
SBP ≥ 140 mmHg or DBP ≥ 90 mmHg (*n* = 102,739)							
	No Head trauma	20,013/20,491 (97.7%)	80,726/82,248 (98.2%)	1		1	
	Head trauma	478/20,491 (2.3%)	1522/82,248 (1.9%)	1.27 (1.14–1.41)	<0.001 *	1.28 (1.16–1.43)	<0.001 *
Fasting blood glucose < 100 mg/dL (*n* = 86,876)							
	No Head trauma	17,144/17,516 (97.9%)	68,224/69,360 (98.4%)	1		1	
	Head trauma	372/17,516 (2.1%)	1136/69,360 (1.6%)	1.30 (1.16–1.47)	<0.001 *	1.32 (1.17–1.48)	<0.001 *
Fasting blood glucose ≥ 100 mg/dL (*n* = 55,679)							
	No Head trauma	10,714/10,995 (97.4%)	43,738/44,684 (97.9%)	1		1	
	Head trauma	281/10,995 (2.6%)	946/44,684 (2.1%)	1.21 (1.06–1.39)	0.005 *	1.23 (1.07–1.41)	0.003 *
Total cholesterol < 200 mg/dL (*n* = 75,303)							
	No Head trauma	14,716/15,096 (97.5%)	58,997/60,207 (98.0%)	1		1	
	Head trauma	380/15,096 (2.5%)	1210/60,207 (2.0%)	1.26 (1.12–1.41)	<0.001 *	1.29 (1.15–1.45)	<0.001 *
Total cholesterol ≥ 200 mg/dL (*n* = 67,252)							
	No Head trauma	13,142/13,415 (98.0%)	52,965/53,837 (98.4%)	1		1	
	Head trauma	273/13,415 (2.0%)	872/53,837 (1.6%)	1.26 (1.10–1.45)	0.001 *	1.26 (1.10–1.45)	0.001 *
CCI scores = 0 (*n* = 85,370)							
	No Head trauma	15,718/16,017 (98.1%)	68,432/69,353 (98.7%)	1		1	
	Head trauma	299/16,017 (1.9%)	921/69,353 (1.3%)	1.41 (1.24–1.61)	<0.001 *	1.43 (1.25–1.63)	<0.001 *
CCI score = 1 (*n* = 24,420)							
	No Head trauma	5532/5683 (97.3%)	18,290/18,737 (97.6%)	1		1	
	Head trauma	151/5683 (2.7%)	447/18,737 (2.4%)	1.12 (0.93–1.35)	0.247	1.16 (0.96–1.40)	0.131
CCI score ≥ 2 (*n* = 32,765)							
	No Head trauma	6608/6811 (97.0%)	25,240/25,954 (97.3%)	1		1	
	Head trauma	203/6811 (3.0%)	714/25,954 (2.8%)	1.09 (0.93–1.27)	0.307	1.13 (0.96–1.33)	0.001 *

Abbreviations: CCI, Charlson Comorbidity Index; SBP, Systolic blood pressure; DBP, Diastolic blood pressure; * Conditional or unconditional logistic regression analysis, Significance at *p* < 0.05 † adjusted for obesity, smoking, alcohol consumption, systolic blood pressure, diastolic blood pressure, fasting blood glucose, total cholesterol, and CCI scores.

## Data Availability

Data is contained within the article or supplementary material.

## Supplementary Materials

The following supporting information can be downloaded at: https://www.mdpi.com/article/10.3390/diagnostics15172171/s1, Table S1: Absolute Risk Increase (ARI) of BPPV Associated with Head Trauma.
