# Supplementary material for: The Association Between Head Trauma and BPPV: A Nested Case-Control Study Using a National Health Screening Cohort
Source: Diagnostics (Basel). 2025 Aug 27;15(17):2171. doi: 10.3390/diagnostics15172171 (PMC12427819; doi:10.3390/diagnostics15172171)
Supplement: Supplementary file 1 [file diagnostics-15-02171-s001.zip › diagnostics-3791509-supplementary.pdf]

S1 Table Absolute Risk Increase (ARI) of BPPV Associated with Head Trauma

Follow-up period	Head trauma (n=19,752	Control (n=79,008)	ARI (%)
Entire follow-up (mean 85.6 month)	4.48% (885/19,752)	3.66% (2,891/79,008)	0.82
1 year	1.33% (263/19,752)	0.67% (529/79,008)	0.66

Abbreviations: BPPV, benign paroxysmal positional vertigo.

Note: Absolute risks and ARIs were calculated using the original Korean National Health Insurance Service–Health Screening Cohort (n = 514,866), in which 20,798 individuals met the head trauma definition. After excluding those with prior BPPV, 19,752 head trauma patients were matched 1:4 with 79,008 controls by age, sex, income, and residential region.
